# Unusual phenotype in 35delG mutation: a case report

**DOI:** 10.1186/s13256-024-04559-3

**Published:** 2024-05-12

**Authors:** Cem Yeral, Lutfu Seneldir, Arzu Hediye Karakoc, Aleyna Sap, Oguz Yilmaz

**Affiliations:** 1 Faculty of Health Sciences, Department of Audiology, İstanbul University-Cerrahpaşa, Istanbul, Türkiye; 2 Faculty of Medicine, Department of Otolaryngology, Istanbul Medipol University, Istanbul, Türkiye; 3 Faculty of Health Sciences, Department of Audiology, Istanbul Medipol University, Istanbul, Türkiye

**Keywords:** GJB2, 35delG mutation, Genetic hearing loss, Unilateral progressive hearing loss

## Abstract

**Background:**

Mutations in the GJB2 gene, which encodes the protein connexin 26 and is involved in inner ear homeostasis, are identified in approximately 50% of patients with autosomal recessive nonsyndromic hearing loss, making it one of the primary causes of prelingual nonsyndromic hearing loss in various populations. The 35delG mutation, one of the most common mutations of the GJB2 gene, usually causes prelingual, bilateral mild to profound, nonprogressive sensorineural hearing loss.

**Case presentation:**

We present an unusual case of an 18-year-old Turkish female with heterozygous 35delG mutation and postlingual, profound-sloping, progressive and fluctuating unilateral sensorineural hearing loss. The phenotype is different from the usual findings.

**Conclusions:**

The 35delG mutation causing hearing loss may not always be reflected in the phenotype as expected and therefore may have different audiologic manifestations.

## Introduction

In developed countries, more than 50% of sensorineural hearing loss (SNHL) cases have a genetic origin; 30% are syndromic, and the remainder are nonsyndromic [1, 2]. Mutations in the gap junction beta 2 protein (GJB2) gene, which encodes the protein connexin 26 and is involved in inner ear homeostasis, are found in approximately 50% of patients with autosomal recessive nonsyndromic hearing loss [2] and is one of the most important causes of prelingual nonsyndromic hearing loss in various populations [3]. The most common mutations of the GJB2 gene, which may vary among different ethnic groups, are 35delG, 167delT and 235delC [4, 5]. The deletion of a single guanine at position 30–35 in the GJB2 gene, known as 35delG, accounts for 70% of mutant alleles [4].

Since 1997, when GJB2 was reported to be the first autosomal recessive gene associated with nonsyndromic SNHL [1], many studies have been published in the literature on the auditory phenotype of mutations in the GJB2 gene [1–3]. However, the type of mutation in the GJB2 gene is also an important factor affecting the auditory phenotype. In general, the hearing loss phenotype of 35delG mutations in the GJB2 gene is prelingual, bilateral, mild to profound, sensorineural and nonprogressive [1, 2, 6, 7]. However, cases with progression and postlingual onset have also been reported [1, 3].

Unlike the SNHL phenotypes observed in most 35delG mutations in the literature, we present a patient with a postlingual, profound-sloping, progressive and fluctuating unilateral SNHL phenotype. We thought that our case report could contribute to the audiological evaluation and diagnosis processes of such unusual cases.

## Case presentation

Informed consent was obtained from the patient’s parent to share the test results and patient information. The patient was an 18-year-old Turkish female. She was born prematurely at 37 weeks of gestation by vaginal delivery without any known complications during pregnancy. Because she was born prematurely, her lungs were underdeveloped. Therefore, she stayed in the incubator for 10 days after her birth and then passed the newborn hearing screening for both ears. At the age of 17 years, her hearing and speech had normally developed. The patient was also diagnosed with epilepsy and rosacea. She was taking sodium valproate (500 mg) and levetiracetam (1000 mg) for epilepsy treatment. There was no known history of other metabolic diseases, physical abnormalities or consanguineous marriages.

### Audiological evaluation

**Fig. 1:**
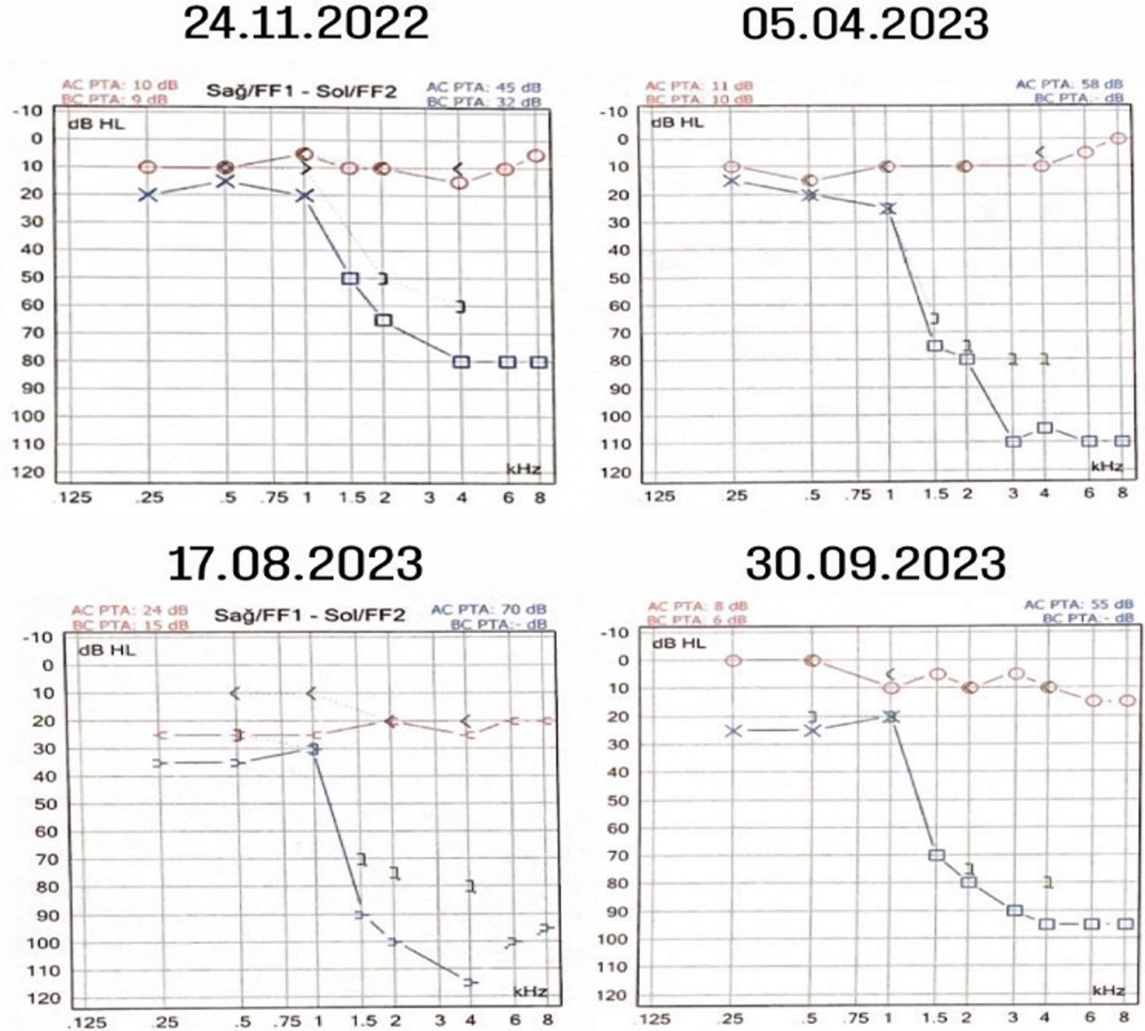
Pure tone audiometry configurations

The patient was admitted to our clinic in November 2022 with a complaint of hearing loss (HL) in her left ear. Hearing evaluation revealed findings consistent with moderate (PTA: 45 dB HL) SNHL with a severe sloping configuration on the left ear (Fig. 1). The second hearing evaluation was performed on April 2023, and the findings were consistent with moderately severe (PTA: 58 dB HL) SNHL with a profoundly sloping configuration on the left ear (Fig. 1). The third hearing evaluation was performed in August 2023, and the findings were consistent with moderately severe (PTA: 70 dB HL) SNHL with a profoundly sloping configuration on the left ear (Fig. 1). A recent hearing evaluation was performed in September 2023, and the findings were consistent with moderate (55 dB HL) SNHL with a profoundly sloping configuration on the left ear (Fig. 1). The speech discrimination scores for the left ear in the four tests were as follows: 64%, 20%, 20% and 44%. As a result of all hearing evaluations, findings compatible with normal hearing were observed for the right ear. A profound sloping configuration was observed in all hearing tests performed on four different dates.

As a result of the immittance assessments, bilateral type A tympanograms were obtained. The acoustic reflex was measured at all frequencies in the right ear but only at 500 Hz in the left ear.

Transient Oto-Acoustic Emission (TEOAE) and Distortion Product Oto-Acoustic Emission (DPOAE) responses were present in the right ear, while no response was detected in the left ear.

**Fig. 2:**
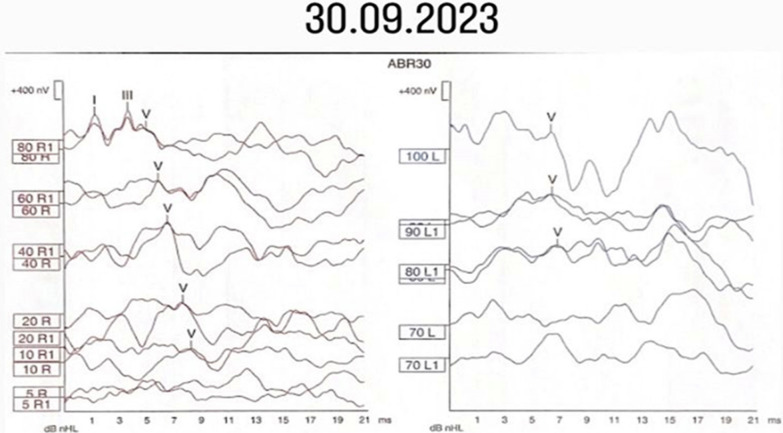
Wave morphologies of the Auditory Brainstem Response test

In the ABR test performed in September 2023, wave V was obtained at 10 dB nHL in the right ear and 80 dB nHL in the left ear (Fig. 2). Wave morphology was distorted in the left ear. Cochlear microphonics were not obtained.

No pathological findings were found as a result of temporal MRI due to the existence of asymmetric hearing loss.

### Genetic evaluation

Whole exome sequencing analysis of DNA isolated from the patient's peripheral blood at the Medical Genetics Laboratory using next-generation sequencing revealed that the "pathogenic" p.G12Vfs*2 (c.35del) frameshift mutation in the GJB2 gene was heterozygous.

## Discussion

Among the nonsyndromic causes of hearing loss, the Connexin 26 (Cx26) gene mutation, encoding the autosomal recessive DFNB1 phenotype, is the most common. The main pathological mechanism of hearing loss caused by GJB2 mutation is still unclear [8]. K + circulation, columnar cell cytoskeletal developmental defects, changes in Ca2 + signaling, ATP release and Ca metabolism, and cochlear macrophages participating in the process of OHC loss are considered among the possible causes [8].

Although 35delG is the most common genotype with a prelingual, bilateral mild to profound, nonprogressive SNHL phenotype [1, 2, 5–7], here, we report an unusual case of a patient with a heterozygous 35delG mutation with a postlingual, profound-sloping progressive and fluctuating unilateral SNHL phenotype.

As a congenital anomaly that has genetic origins, most of the cases are postlingual SNHL [9]. Wingard and Zhao suggested that postlingual SNHL may result from impaired active cochlear amplification due to Cx26 deficiency [10]. In the present case, the patient's hearing complaints appeared only in the left ear at the age of 17 years.

Although 35delG mutations are generally known to affect both ears, Lee *et al.* reported that individuals with heterozygous mutations in GJB2, the gene associated with 35delG mutations, showed more asymmetric SNHL than those with homozygous mutations [7]. Additionally, the degree of hearing loss in individuals with GJB2 mutations can be variable, and the degree of hearing loss associated with all reported genotypes has not been demonstrated [11]. Hilgert *et al.* reported that hearing loss ranges from mild to moderate (least frequent) or severe to profound (most frequent) and that in addition to familial factors, this phenotypic variation can be explained by the influence of environmental factors, modifier genes and/or other connexin genes [12]. Salvinelli *et al.* reported that the residual audiogram configuration in 35delG homozygous patients was more common, whereas the gently sloping configuration was more common in 35delG heterozygous individuals [6]. Marlin *et al.* documented progressive hearing loss in 22% of 256 subjects and concluded that although DFNB1 hearing loss is mostly stable, it can also be progressive [13]. The first three hearing assessments of the patient demonstrated that the SNHL in the left ear had progressed and that the right ear was stable. At the fourth evaluation, a slight improvement in the left ear hearing threshold was observed, and this finding was interpreted as fluctuating hearing loss. To our knowledge, there are no patients with fluctuating SNHL due to the 35delG mutation.

In some of the case reports on GJB2, OAE and ABR tests were also performed [14]. In the present case, TEOAE and DPOAE responses were present in the right ear but not in the left ear. According to the ABR results, wave V was obtained at 10 dB nHL in the right ear and 80 dB nHL in the left ear. Cochlear microphonics were not obtained bilaterally. The OAE and ABR findings were consistent with the behavioral thresholds. Given that temporal MRI was also normal, the results allowed us to exclude the possibility of an auditory neuropathy spectrum disorder or any retrocochlear pathology.

Mutations in the GJB2 gene may cause skin abnormalities in addition to hearing loss. In the literature, these mutations may be responsible for keratitis- ichthyosis- deafness (KID) syndrome, palmoplantar keratoderma (PPK) syndrome, Vohwinkel syndrome, and extensive hyperkeratotic lesions in the skin [15]. Our patient was also diagnosed with rosacea. There is no evidence in the literature showing that mutations in this gene are associated with rosacea disease. Therefore, it is not clear whether this disease is related to the GJB2 mutation.

## Conclusion

We described an unusual case of a heterozygous 35delG mutation with postlingual, profound-sloping progressive and fluctuating unilateral SNHL with a phenotype different from common findings. The 35delG mutation causing hearing loss may not always be reflected in the phenotype as expected and may have different audiologic manifestations. In patients with a history of unilateral SNHL that occurs in a short period of time, a detailed anamnesis of the importance of imaging methods, genetic examination and audiologic tests in the diagnostic process should be performed. Genetic counseling should be provided to individuals with genetic hearing loss and their families. Regular follow-up of these individuals is very important to provide appropriate interventions for hearing loss.

## Data Availability

Data sharing is not applicable to this article, as no datasets were generated or analyzed during the current study.
